# Beclin 1 Phosphorylation – at the Center of Autophagy Regulation

**DOI:** 10.3389/fcell.2018.00137

**Published:** 2018-10-12

**Authors:** Manoj B. Menon, Sonam Dhamija

**Affiliations:** ^1^Institute of Cell Biochemistry, Hannover Medical School, Hannover, Germany; ^2^Division of Cancer Research, Department of Thoracic Surgery, Medical Center – University of Freiburg, Freiburg, Germany; ^3^Division of RNA Biology and Cancer, German Cancer Research Center, Heidelberg, Germany

**Keywords:** autophagy, BECN1, phosphorylation, PI3K, kinase, ATG, beclin

## Abstract

Autophagy is a tightly regulated catabolic process wherein cells under stress sequester cytosolic constituents like damaged proteins and organelles in double-membrane vesicles called autophagosomes. The autophagosomes degrade their cargo by lysosomal proteolysis generating raw materials for the biosynthesis of vital macromolecules. One of the initial steps in the assembly of autophagosomes from pre-autophagic structures is the recruitment and activation of the class III phosphatidylinositol 3-kinase complex consisting of Beclin 1 (BECN1), VPS34, VPS15, and ATG14 proteins. Several pieces of evidence indicate that the phosphorylation and ubiquitination of BECN1 at an array of residues fine-tune the responses to diverse autophagy modulating stimuli and helps in maintaining the balance between pro-survival autophagy and pro-apoptotic responses. In this mini-review, we will discuss the importance of distinct BECN1 phosphorylation events, the diverse signaling pathways and kinases involved and their role in the regulation of autophagy.

## Introduction

**FIGURE 1:**
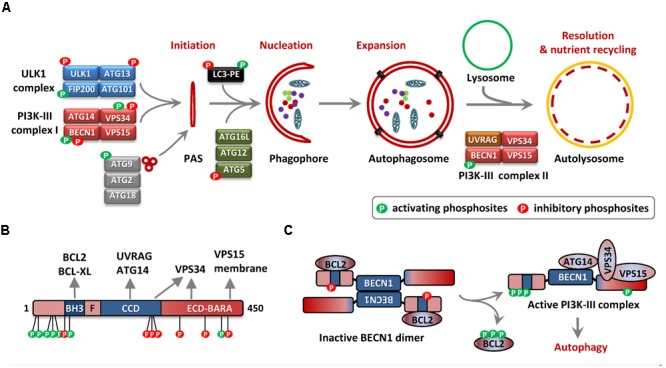
Autophagy and Beclin 1 (BECN1) phosphorylation. **(A)** The scheme depicts the phases of autophagosome assembly from initiation to nutrient recycling. The steps include the structural transformation from the pre-autophagosomal structure (PAS) to phagophore to autophagosomes, culminating in the fusion of autophagosomes with lysosomes facilitating the degradation of their contents in autolysosomes. The regulatory protein complexes involved are depicted with their components and the presence of regulatory phosphorylation event/events are shown. **(B)** Primary structure of BECN1 showing the BCL2/BCL-XL binding BH3 motif (residues 105–130), flexible helical domain (F, residues 141–171), and the central coiled coil domain (CCD, residues 175–265). Evolutionary conserved domain (residues 248–337) and β/α-repeated, autophagy-related (BARA) domain (265–450aa) is represented together as ECD-BARA (Mei et al., 2016). The approximate locations of pro-autophagy (green) and inhibitory (red) phosphorylation sites are shown. The contributions of the different domains to complex formation with interactors are also indicated. **(C)** Phosphorylation-dependent conversion of inactive BECN1 homodimer/BCL2-complex to an active PI3K-III complex is depicted. The STK4-mediated BECN1-BH3 domain phosphorylation (negative regulator of autophagy), triple phosphorylation of BCL2 which releases BCL2 from BECN1, representative phosphorylation events in the N-terminal domain (NTD) promoting BECN1-BCL2 dissociation as well as activating the PI3K-activity are presented (positive regulation).

Macro-autophagy, usually referred to as “autophagy” is a catabolic process by which cells subject cytosolic components for lysosomal degradation and recycling, often in response to metabolic stress or nutrient starvation (Bento et al., 2016). In response to the metabolic needs of the cells, autophagy is initiated by the concerted action of the ATG1 (autophagy related 1)/ULK1 (Unc-51 like autophagy activating kinase-1) complex and the PI3K-III complex. This leads to the nucleation of phagophores which engulf intracellular cargo generating double-membranous structures called autophagosomes, which eventually fuse with lysosomes to form autolysosomes wherein the contents are degraded to release amino acids and other metabolites (**Figure 1A**; Bento et al., 2016). Many cancer cells display high rates of basal autophagy, providing them resistance to metabolic stress, which in turn makes autophagy a target for anti-cancer intervention (Bhat et al., 2018). Even though primarily a pro-survival process, excessive “self-eating” could be deleterious to the cells and hence the process is tightly regulated and coupled to the energy and nutrient necessities of the cells. Different nutrient sensing pathways convey signals to the autophagy machinery, phosphorylating autophagy related gene-products and regulating diverse phases of autophagosome formation and resolution. Being the master nutrient sensor, the mTORC1 (mammalian target of rapamycin complex 1) signaling is the primary regulator of autophagy initiation in mammalian cells (Kim and Guan, 2015; Rabanal-Ruiz et al., 2017). Under nutrient rich conditions mTORC1 phosphorylates ULK1 and ATG13 in the ULK1 complex effectively suppressing ULK1 activity and autophagy. Loss of mTORC1 activity during amino-acid and nutrient starvation releases this break on ULK1 activation and initiates autophagy. AMPK (5′ adenosine monophosphate-activated protein kinase), a kinase which is activated in response to energy-depletion in the cell also regulates the activity of the ULK1 complex. mTORC1-dependent suppression and AMPK-mediated activation of the ULK1 complex are among the most studied mechanisms coupling nutrient sensing to autophagosome biogenesis. However, several studies have identified the PI3K-III (phosphatidylinositol 3-kinase, class III) complex as an equally important signaling hub which fine tunes the autophagy-flux in response to diverse signals and balances autophagy with apoptosis. Here, we will summarize the current literature on the regulation of autophagy by the phosphorylation of the PI3K-III regulatory subunit BECN1 (Beclin-1) (**Table 1**).

**Table 1:** Beclin 1 (BECN1) phosphorylation sites, their domain localization, effect on autophagy and the kinases involved.

No.	Kinase	Phospho-site (hBECN1)	BECN1 domain	Effect on autophagy	Reference
1	ULK1	S15, S30	NTD	Positive	Russell et al., 2013; Park et al., 2018
2	PGK1	S30	NTD	Positive	Qian et al., 2017
3	DAPK3	S90	NTD	Positive	Fujiwara et al., 2016
4	MAPKAPK2/3	S90	NTD	Positive	Wei et al., 2015
5	CAMKII	S90	NTD	Positive	Li et al., 2017
6	AMPK	S93, S96	NTD	Positive	Kim et al., 2013
	STK4	T108	BH3 motif	Negative	Maejima et al., 2013
7	DAPK1	T119	BH3 motif	Positive	Zalckvar et al., 2009b
8	DAPK2	T119	BH3 motif	Positive	Shiloh et al., 2018
9	ROCK1	T119	BH3 motif	Positive	Gurkar et al., 2013
10	FAK	Y233	CCD	Negative	Cheng et al., 2017
11	AKT1	S234, S295	CCD, ECD-BARA	Negative	Wang et al., 2012
12	EGFR	Y229, Y233, Y352	CCD, BARA	Negative	Wei et al., 2013
13	BCR-ABL	Y233, Y352	CCD, BARA	Negative	Galluzzi et al., 2018
14	AMPK	T388	BARA	Positive	Zhang et al., 2016
15	CSNK1G2	S409	BARA	Negative	Sun et al., 2015

## BECN1 – at the Cross-Roads of Autophagy and Apoptosis

Beclin 1 was originally identified as a yeast two-hybrid interaction partner of the anti-apoptotic protein – BCL2 (Liang et al., 1998). Soon after that, it was described as the mammalian homolog of yeast Atg6, involved in autophagy and tumor suppression, often deleted in ovarian and breast cancer (Aita et al., 1999; Liang et al., 1999). Consistent with its dual role in the processes of autophagy and apoptosis, homozygous deletion of *Becn1* in mice results in early embryonic lethality, while *Becn1^+/-^* mice are prone to spontaneous and virus-induced tumorigenesis (Qu et al., 2003; Yue et al., 2003). BECN1 associates with PIK3C3/ VPS34 (vacuolar sorting protein-34), a class III phosphoinositide kinase catalytic subunit and the regulatory subunit VPS15/PIK3R4 to form a highly regulated complex which promotes PtdIns-3P (phosphatidylinositol 3-phosphate) generation. AMBRA1 (activating molecule in BECN1 regulated autophagy) anchors the BECN1-VPS34 complex to dynein light chains and the phosphorylation of AMBRA-1 by ULK1 is necessary for releasing the complex from the microtubule motor, facilitating ER (endoplasmic reticulum) localization and phagosome biogenesis (Gao et al., 2016). Multiple BECN1 interacting proteins are recruited to this minimal unit, including ATG14/Barkor (BECN1-associated autophagy-related key regulator), UVRAG (UV-radiation resistance associated gene), and Rubicon (RUN and cysteine rich domain containing BECN1 interacting protein). However, a complex consisting of VPS34, BECN1, ATG14 and the membrane anchoring VPS15 is the essential unit necessary for the localized generation of PtdIns-3P facilitating initiation of autophagy (Matsunaga et al., 2010). UVRAG has been shown to associate with BECN1 in a mutually exclusive manner with ATG14, generating a distinct PI3-kinase complex which functions in vacuolar protein sorting pathway, rather than autophagy (Itakura et al., 2008). Rubicon is an inhibitor of VPS34 function which specifically associates with a sub-fraction of UVRAG-containing complexes (Matsunaga et al., 2009; Zhong et al., 2009). Even though there are three distinct VPS34-BECN1 regulatory units, sufficient evidence exists in the literature which suggests active cross-talk between these membrane remodeling complexes in vesicular trafficking and autophagosome maturation (Liang et al., 2008; Matsunaga et al., 2009; Zhong et al., 2009; Wu et al., 2018).

Beclin 1 is a multi-domain protein with an N-terminal BCL2 homology (BH)-3 domain, a central coiled-coil domain (CCD), an evolutionarily conserved domain (ECD) and an overlapping C-terminal β-α-repeated, autophagy-specific (BARA) domain (**Figure 1B**). The central CCD can dimerize in antiparallel orientation to generate an inactive BECN1 homodimer or facilitate complex formation with ATG14 or UVRAG, leading to an active PI3-kinase complex. The interaction between BECN1 BH3 domain and BCL2/BCL-XL stabilizes CCD-mediated BECN1 dimers, effectively suppressing autophagosome biogenesis (Pattingre et al., 2005). This forms the primary mechanism of BECN1-dependent cross-talk between apoptosis and autophagy and is target for regulation by post-translational modifications (PTMs) (Levine et al., 2008). JNK1 (c-Jun N-terminal kinase / MAPK8) or ERK1/2 (MAPK1/3) – mediated phosphorylation of BCL2 upon starvation induces the dissociation of BECN1-BCL2 complex, facilitating heterodimerization of BECN1 with ATG14 and activation of autophagy (Tang et al., 2010; Song et al., 2018b; **Figure 1C**). Consistently, over-expression of a BCL2-binding deficient mutant of BECN1 is sufficient to induce an autophagic response, even under nutrient-rich conditions (Pattingre et al., 2005). Thus the release of BECN1 from BCL2/BCL-XL proteins seems to be an essential step for initiating the autophagic response. This is further confirmed by the fact that BH3-only domain proteins like BAD as well as the BH3-mimetic compound ABT-737 competitively displace BECN1 from the BH3-binding grooves on BCL2/BCL-XL and activate autophagosome formation (Maiuri et al., 2007a,b). The BH3-mimetics are inhibitors of the anti-apoptotic action of the BCL2 family proteins and are strong inducers of apoptosis (Oltersdorf et al., 2005). However, BECN1 association doesn’t affect the anti-apoptotic functions of BCL2/BCL-XL and distinct pools of BCL2 localized to separate sub-cellular membranes seems to regulate autophagy and apoptosis (Ciechomska et al., 2009; Chang et al., 2010). Moreover, the ECD, which is crucial for recruiting VPS34 is necessary for inducing autophagy as well as tumor suppression, suggesting that the tumor suppressor function of BECN1 is coupled to the lipid kinase function, rather than BCL2 association (Furuya et al., 2005). However, the expression of a BCL2-binding deficient BECN1 mutant not only promoted autophagy, but also enhanced basal and starvation-induced apoptosis in MCF7 cells. Interestingly, the pro-apoptotic effect is abrogated upon siRNA mediated depletion of ATG5, suggesting that the apoptotic response is downstream to autophagy (Pattingre et al., 2005). “Autophagic-death” has been much debated and has already been discounted as a misnomer and autophagy is now widely being accepted as a cytoprotective process, which helps in adaptation against stress (Levy et al., 2017). However, in several cases, the components of the autophagy machinery contributes to the cell death process and the term “autophagy-dependent cell death” has been coined to describe such events of regulated cell death (Galluzzi et al., 2018; Lima et al., 2018). For example, ferroptosis – a form of programmed necrosis requires the degradation of iron storage proteins by autophagy, making it a type of autophagy-dependent cell death (Gao et al., 2016; Hou et al., 2016). In addition to this, there is a fine balance between the pro-survival autophagic response and the apoptosis response under diverse conditions. For example, inhibition of autophagy by ATG5-depletion leads to stronger apoptotic cell killing in response to staurosporine in the H4 glioma cells (Xu et al., 2013). BECN1 and its interactors may act as mediators of this cross-talk. Caspase and calpain mediated cleavage of BECN1 and AMBRA1 seems to be a mode of suppressing the autophagic response (Cho et al., 2009; Luo and Rubinsztein, 2010; Russo et al., 2011; Pagliarini et al., 2012). The embryonic lethality in *Ambra1^-/-^* mice is associated with enhanced apoptotic cell death, in addition to signs of impaired autophagy (Fimia et al., 2007). BECN1 can also be involved in autophagic suppression of apoptosis. TRAIL (tumor necrosis factor related apoptosis inducing ligand), induces RIPK1 (receptor interacting S/T kinase-1)-dependent BECN1 expression, autophagy and apoptotic cell death (Yao et al., 2015). Under such conditions, TRAIL-mediated autophagy suppresses apoptosis by inducing CASP8 degradation and autophagy inhibition is a prerequisite for efficient cell death response (Hou et al., 2010). A recent study also reported an autophagy-apoptosis switch in the context of combinatorial chemotherapy, wherein the dephosphorylation of BECN1 at S234/S295 leads to enhanced caspase-cleavage and suppression of autophagy (Song et al., 2018a).

## Regulation of Autophagy By BECN1 Phosphorylation

As indicated previously, the release of BECN1 from BCL2/BCL-XL association at the ER-membrane is a prerequisite for its heterodimerization with ATG14 and formation of a functional PI3K-III complex (Chang et al., 2010). Confirming the *in vitro* observations, knock-in mice harboring a non-phosphorylatable mutant of BCL2 (T69A/S70A/S84A) are deficient in exercise-induced myocyte autophagy *in vivo* (He et al., 2012). Moreover, the recent analysis of a *Becn1^F121A/F121A^* knock-in mouse model, wherein a BH3 domain mutation abrogates BECN1-BCL2 interaction, revealed constitutively increased basal autophagy (Rocchi et al., 2017; Fernandez et al., 2018). Interestingly, the F121A knock-in mice displayed enhanced life span, health span as well as improved cognitive function in a mouse model of Alzheimer’s disease (Rocchi et al., 2017; Fernandez et al., 2018). While ERK/JNK-mediated phosphorylation of BCL2 seems to be effective in abrogating BECN1-BCL2 interaction, such phosphorylation events are not known for BCL-XL, suggesting the existence of other modes of regulation. Over the years, a number of kinases have been shown to phosphorylate the N-terminal region of BECN1, thus affecting the association of the BH3-domain with the BCL2/BCL-XL proteins (**Table 1**). In one of the earliest reports, DAPK1 (death-associated protein kinase-1) was shown to phosphorylate the BH3-domain residue T119 and consequently abrogate BECN1-BCL2/BCL-XL interaction, facilitating autophagy initiation upon serum deprivation (Zalckvar et al., 2009a,b). BECN1-T119 is also targeted by the closely related kinase DAPK2 as well as ROCK1 (Rho-associated coiled-coil containing protein kinase-1), with a similar outcome (Gurkar et al., 2013; Shiloh et al., 2018). A deviation from the general theme of autophagy stimulation by PTM at the BH3-domain interface also exists. STK4 (serine/threonine kinase-4)/Mst1 (mammalian STE20-like protein kinase 1), a proapoptotic kinase stabilizes BECN1-BCL2/BCL-XL association by phosphorylating at BECN1-T108, thus inhibiting autophagy in cardiomyocytes (Maejima et al., 2013; **Figure 1C**). Interestingly, T108 phosphorylation also leads to efficient sequestration of BCL2/BCL-XL by BECN1, activating BAX-mediated apoptosis. This finding is in contrast to other studies which show that anti-apoptotic functions of BCL2 are not compromised by BECN1 and possibly indicates a BECN1-T108 and/or cardiomyocyte-specific effect (Ciechomska et al., 2009; Chang et al., 2010).

The ATG14-BECN1-VPS34 complex formation is not sufficient for initiating autophagy and this complex needs to be activated by ULK1 (or ULK2) mediated S15 phosphorylation to generate a PI3K-III complex proficient in PtdIns-3P production (Russell et al., 2013). ULK1-mediated BECN1-S15 phosphorylation also targets UVRAG-associated complexes, contributing to autophagosome maturation (Russell et al., 2013). Recent evidences indicate a role for S15 phosphorylation in promoting the interaction of BECN1 with the ubiquitin ligase PARK2/Parkin, facilitating translocation of PARK2 to damaged mitochondria during mitophagy (Kumar and Shaha, 2018). In addition to S15, ULK1/2 also phosphorylates BECN1 at S30, exclusively in the ATG14-associated complex to positively regulate early stages of autophagosome biogenesis (Park et al., 2018). Interestingly, a glycolytic enzyme – PGK1 (phosphoglycerate kinase 1) also directly phosphorylates BECN1-S30 leading to enhanced VPS34 activity and autophagy, in response to glutamine deprivation or hypoxia (Qian et al., 2017). Glutamine deprivation induced acetylation of PGK1 is necessary for BECN1 association and phosphorylation. Even though a very attractive idea, whether PGK1 is directly functioning as a protein kinase phosphorylating BECN1-S30 or just indirectly promote ULK1/2-mediated S30 phosphorylation is a debatable question (Park et al., 2018).

AMPK, one of the cellular energy sensors also participates in control of autophagy by direct phosphorylation of BECN1. AMPK-mediated phosphorylation of BECN1 at S93 and S96 is essential for activating the pro-autophagic VPS34-BECN1-ATG14 complex in response to glucose starvation (Kim et al., 2013). Enhanced phosphorylation at S96 is also observed during ethanol-induced autophagy and is suppressed upon siRNA mediated depletion of AMPK (Hong-Brown et al., 2017). Interestingly, recent evidence indicates a role for AMPK-mediated S93/96 phosphorylation of BECN1 in the regulation of ferroptosis, a form of autophagy-dependent cell death (Song et al., 2018b). Another AMPK target site is T388 in the C-terminal region of BECN1. Interestingly, T388 phosphorylation not only enhances the association of BECN1 with the VPS34-ATG14-VPS15 complex, but also reduces BECN1-BCL2 complex formation and BECN1 dimerization (Zhang et al., 2016). The structure of BECN1 C-terminus displays a unique membrane binding fold with 3 structural repeating units consisting of central helices, short strands and loops denoted as the BARA domain (Huang et al., 2012; Noda et al., 2012). Recent *in vitro* studies also suggest a role for the BARA domain residues in efficient BECN1 homodimerization mediated by ECD (Ranaghan et al., 2017). The third unit (aa387–aa444) of the BARA domain harbors the T388 site in a flexible loop, followed by a region (aa425–aa450) shown to be required for VPS15-dependent membrane localization (Huang et al., 2012; Fogel et al., 2013). T388 phosphorylation may interfere with BECN1 dimerization or enhance its membrane association and thus indirectly modulate BCL2 binding at the N-terminal BH3 domain. More *in vitro* studies and structural analysis of full length BECN1 in dimer form and in complex with its interaction partners will be necessary to answer the open questions regarding the homodimer-heterodimer switch.

Beclin 1-S90 and S93 residues were originally identified as ATG14-dependent phosphorylation sites induced during starvation-induced autophagy and valinomycin-induced mitophagy (Fogel et al., 2013). While AMPK seems to be the only kinase phosphorylating at S93, S90 is a target for many kinases. S90 phosphorylation is strongly induced upon amino-acid starvation or treatment with PP2A (protein phosphatase 2A)-inhibitor okadaic acid and is necessary for the activation of autophagy (Wei et al., 2015; Fujiwara et al., 2016). Starvation of mice also induces BECN1-S90 phosphorylation in skeletal muscle, indicating the *in vivo* relevance of this modification (Fujiwara et al., 2016). Interestingly, the SAPK (stress-activated protein kinase) pathways consisting of JNKs and the p38MAPKs converge on the BCL2-BECN1 axis to regulate autophagy. While JNK1 phosphorylates BCL2, inducing the dissociation of the BECN1-BCL2 complex, the p38-downstream kinases MK2 and MK3 directly phosphorylates BECN-S90 activating the lipid kinase activity of the BECN1-VPS34-ATG14 complex, promoting autophagy (Wei et al., 2015). A non-phosphorylatable BCL2 mutant prevents BECN1-S90 phosphorylation, suggesting that the removal of steric blockade by BCL2 is required for phosphorylation by MK2/3 (Wei et al., 2015). DAPK3 is another kinase which has been proposed to be a BECN1-S90 kinase and siRNA mediated depletion of DAPK3 suppresses starvation and PP2A-inhibition induced BECN1-S90 phosphorylation in HeLa cells (Fujiwara et al., 2016). In response to calcium stimulation, CaMKII (Calcium/calmodulin-dependent protein kinase II) mediates S90 phosphorylation, which facilitates K63-ubiquitination-dependent autophagic degradation of Id1/2 (inhibitor of differentiation-1/2) promoting neuroblastoma differentiation (Li et al., 2017).

Most of the phosphorylation sites on the C-terminal half of BECN1 are inhibitory in nature except for AMPK-dependent T388 phosphorylation (**Figure 1B** and **Table 1**). Akt mediated phosphorylation at S234 and S295 leads to the suppression of BECN1 functions in autophagy and tumor suppression (Wang et al., 2012). In an interesting cross-talk between autophagy and cytoskeleton, the phosphorylated BECN1 is sequestered by 14-3-3 proteins, which anchors them to vimentin intermediate filaments, preventing autophagosome formation (Wang et al., 2012). Another related S/T kinase, CK1γ2 / CSNK1G2 (Casein kinase 1 gamma 2) targets BECN1-S409 and this phosphorylation enhances BECN1 acetylation at K430/K437 promoting interaction with Rubicon (Sun et al., 2015). Rubicon binding to UVRAG-associated VPS34-BECN1 complexes suppresses vesicular transport and inhibits autophagosome maturation (Matsunaga et al., 2009; Sun et al., 2015).

In addition to the S/T-kinases, there is also tyrosine phosphorylation-dependent suppression of BECN1 function in autophagy. FAK/PTK2 (focal-adhesion kinase/protein tyrosine kinase-2) mediated phosphorylation at BECN1-Y233 suppresses cardiomyocyte autophagy and promotes BECN1-dependent cardiomyocyte hypertrophy *in vivo* (Cheng et al., 2017). At the molecular level, Y233 phosphorylation seems to suppress ATG14-interaction (Cheng et al., 2017). Interestingly, autophagy is in turn involved in the degradation of focal-adhesions, with effect on cancer cell metastasis and FAK-mediated suppression of autophagy could be part of a feed-back loop (Sharifi et al., 2016; Kawano et al., 2017; Wang et al., 2018). In the context of host-pathogen interaction, suppression of autophagy by *Salmonella* seems to depend on FAK-dependent mTOR activation, but a direct role for FAK-mediated BECN1 phosphorylation could also be relevant (Owen et al., 2014). EGFR (human epidermal growth factor receptor / HER1) is another tyrosine kinase involved in autophagy regulation by BECN1 phosphorylation. EGFR activation by oncogenic mutations induces phosphorylation of BECN1 at Y229, Y233, and Y352, which suppress autophagy and enhance tumorigenesis in a NSCLC (non-small cell lung carcinoma) xenograft model (Wei et al., 2013). This multisite tyrosine phosphorylation alters BECN1-interactome by promoting homodimerization, BCL2 association and Rubicon binding, while inhibiting the BECN1-VPS34 complex formation (Wei et al., 2013). In a parallel study, HER2 (human epidermal growth factor receptor 2) was shown to form an EGFR/HER2-inhibitor sensitive complex with BECN1 at the plasma membrane resulting in HER2 phosphorylation, Akt activation and suppression of autophagy (Han et al., 2013). In EGFR/HER2-inhibitor resistant breast cancer cells, there is enhanced autophagy in response to the inhibitor, which can be switched to apoptosis by BECN1 depletion (Han et al., 2013). Considering the similarities between EGFR and HER2, HER2-dependent suppression of autophagy could also involve direct BECN1 phosphorylation. In CML (chronic myelogenous leukemia), the constitutive active chimeric tyrosine kinase BCR-ABL is involved in phosphorylating Y233 and Y352 residues, keeping autophagy under check and promoting leukemogenesis (Galluzzi et al., 2018). However, upon treatment with receptor tyrosine kinase inhibitors, there is upregulation of pro-survival autophagy, which makes co-targeting of autophagy an effective therapeutic strategy (Levine et al., 2008; Tang et al., 2010).

## Other PTMs Regulating BECN1

Beclin 1 is a signaling hub in the context of autophagy as well as vesicular sorting pathway and is subjected to a wide array of PTMs. In addition to 23 phosphorylation sites, proteomic data publicly available in the PhosphoSitePlus^®^ database shows the presence of nine ubiquitination and three acetylation sites on human BECN1 (Hornbeck et al., 2015). Much like the phosphorylation sites, the best analyzed ubiquitination site is in the BH3 domain and TRAF6-mediated K63-linked ubiquitination at BECN1-K117 prevents BCL2/BCL-XL association, facilitating TLR4 (toll-like receptor 4) induced autophagy (Shi and Kehrl, 2010a,b; Min et al., 2018). TRIM50 (Tripartite Motif Containing 50) is another ubiquitin ligase which adds pro-autophagic K63-linked ubiquitin chains to BECN1, but the exact lysine residues targeted by this ligase are not known (Fusco et al., 2018). During starvation-induced autophagy, K63-linked poly-ubiquitination at BECN1-K347 is necessary for efficient VPS34 association and activation (Xia et al., 2013). This modification is mediated by the CUL4-DDB1 E3-ubiquitin ligase complex, with BECN1 interactor AMBRA1 as one of its components (Xia et al., 2013).

An interesting mechanism of BECN1-ubiquitination dependent suppression of autophagy emerged from the pharmacology front, when a screen for autophagy inhibitors identified a small molecule named spautin-1, which causes ubiquitination and proteasomal-degradation of BECN1 (Liu et al., 2011). Depletion of BECN1 leads to the degradation of ATG14 and destabilization of the pro-autophagic ATG14-BECN1-VPS34 complex, suppressing autophagy (Thoresen et al., 2010; Liu et al., 2011). The targets of spautin-1 are the ubiquitin specific proteases USP10 and USP13, but the ubiquitin ligases and the specific ubiquitination sites involved in degradative BECN1 ubiquitination in this context is not known so far (Liu et al., 2011). Ataxin-3, another deubiquitinating enzyme is involved in deubiquitinating K48-linked poly-ubiquitin chains from BECN1-K402, thus stabilizing BECN1 and promoting starvation-induced autophagy (Ashkenazi et al., 2017). Inhibition of the chaperone protein HSP90 (heat-shock protein-90) also induces K48-linked poly-ubiquitination and degradation of BECN1, suppressing TLR-mediated autophagy (Xu et al., 2011). Ring family ubiquitin ligase RNF216 (ring-finger protein-216) is another protein which suppresses autophagy by inducing K48-linked poly-ubiquitination and degradation of BECN1 in TLR-stimulated macrophages and colon cancer (Xu et al., 2014; Wang et al., 2016). Although the ubiquitin ligase NEDD4 (neural precursor cell expressed developmentally down-regulated protein 4) was shown to inhibit autophagy by K11-linked poly-ubiquitination and degradation of BECN1, recent evidences suggest a pro-autophagic role for this protein (Platta et al., 2012; Pei et al., 2017; Sun et al., 2017). However, K11-ubiquitination of BECN1 at K437 is indeed a negative regulator of BECN stability and is counteracted by the ER-tail-anchored ubiquitin specific protease USP19 (Jin et al., 2016). Interestingly, ubiquitination seems to be a very important means of fine-tuning autophagic response, especially in the case of TLR/immune signaling and bacterial autophagy. CUL3-KLHL20 E3-ubiquitin ligase complex mediated ubiquitination of several ATG proteins including BECN1 and subsequent proteasomal-degradation is involved in the termination of autophagy (Liu et al., 2016).

In addition to phosphorylation and ubiquitination, S409 phosphorylation primed acetylation at K430/K437 is an important regulatory mechanism inducing association of BECN1with Rubicon and suppressing autophagy (Sun et al., 2015). While S409 phosphorylation enhances the interaction of BECN1 with the acetyl transferase p300 facilitating acetylation, SIRT1 seems to be the specific deacetylase targeting BECN1 (Sun et al., 2015). Even though there is no direct proof for methylation of BECN1, the consensus sequences surrounding S90, identify R87 as a possible regulatory methylation target (Fogel et al., 2013). Moreover, BECN1 is also subjected to O-GlcNAcylation, but the target sites and functional relevance of this modification has not been investigated in detail (Matsunaga et al., 2010).

## BECN1 Modifications and Functions: Challenges Ahead

There is mass-spectrometric evidence for PTMs at almost 30% of all the modifiable residues (S/T/Y/K) on BECN1^1^. Regulatory sites are uniformly spread throughout the length of the protein and analysis of missense mutations in pathologies like HCC (hepatocellular carcinoma) has identified large scale changes affecting relevant residues (Levy et al., 2017; Wu et al., 2018). The functional relevance of these sites, especially ubiquitination sites are unexplored so far. Much of our understanding on BECN1modifications and autophagy in general is deduced from non-physiological setting in cultured cells. A comparative analysis of autophagy in response to distinct stimuli like amino-acid starvation, glucose starvation, protein aggregation, hypoxia and TLR-stimulation will be useful to make note of basic and stimulus-specific factors involved. This could also be helpful in demystifying the signaling aspect of autophagy-dependent phenotypes in animal models. In addition to PTMs, transcriptional upregulation of BECN1 is also often associated with enhanced autophagy in response to diverse stimuli (Matsunaga et al., 2009; Gao et al., 2016; Song et al., 2018b). While the initiation and termination of autophagy depends on dynamic PTMs, the long-term effects are mediated by transcription regulatory circuits. The literature on basal autophagy signaling, responses to therapy and related phenotypes in cancer are rather scattered and incomprehensible, due to overlapping transcriptional and post-translational regulatory networks. There is a need to revisit some of the early findings related to autophagy-dependent cell death, especially to distinctly separate this from defective autophagy-induced apoptosis.

Even though the functions of BECN1 can be simply represented as regulation of vacuolar protein sorting and autophagy, relevance of this signaling molecule encompass immunity, metabolism, and cancer (Zhong et al., 2016). Modified themes of BECN1 functions in these membrane associated complexes have emerged, which makes it a target for therapeutic intervention in pathologies ranging from cancer to Alzheimer’s disease (Fimia et al., 2007; Xu et al., 2013; Rocchi et al., 2017). In addition, there are BECN1-independent mechanisms of autophagy and autophagy-independent roles for BECN1 in cancer (Scarlatti et al., 2008; Smith et al., 2010; Elgendy et al., 2014). The biochemical analysis of BECN1 and associated factors are constantly changing our understanding on the structure-function correlation of different BECN1 domains. For example, a recent study suggests the involvement of C-terminal BARA domain in BECN1 dimerization and BCL2 interaction, which may argue against the antiparallel homodimer model represented in **Figure 1C** (Ranaghan et al., 2017). The field of BECN1 research will be entering its third decade soon. Persistent work from several groups, integrating diverse biological systems have revealed a lot, but the complexities of this multifaceted signaling molecule is far from being completely understood.

## Author Contributions

Both authors contributed to the drafting and revision of the manuscript and approved it for publication.

## Conflict of Interest Statement

The authors declare that the research was conducted in the absence of any commercial or financial relationships that could be construed as a potential conflict of interest.

## Footnotes

1 http://www.phosphosite.org/
